# Divide-and-conquer Tournament on Social Networks

**DOI:** 10.1038/s41598-017-15616-x

**Published:** 2017-11-14

**Authors:** Jiasheng Wang, Yichao Zhang, Jihong Guan, Shuigeng Zhou

**Affiliations:** 1 Department of Computer Science and Technology, Tongji University, 4800 Cao’an Road, Shanghai, 201804 China; 2 Key Laboratory of Embedded System and Service Computing (Tongji University), Ministry of Education, Shanghai, 200092 China; 3 School of Computer Science, Fudan University, 220 Handan Road, Shanghai, 200433 China; 4 Shanghai Key Laboratory of Intelligent Information Processing, Shanghai, 200433 China

## Abstract

In social gaming networks, previous studies extensively investigated the influence of a variety of strategies on reciprocal behaviors in the prisoner’s dilemma game. The studied frameworks range from the case that an individual uniformly cooperates or defects with all social contacts, to the recently reported divide-and-conquer games, where an individual can choose a particular move to play with each neighbor. In this paper, we investigate a divide-and-conquer tournament among 14 well-known strategies on social gaming networks. In the tournament, an individual’s fitness is measured by accumulated and average payoff aggregated for a certain number of rounds. On the base of their fitness, the evolution of the population follows a local learning mechanism. Our observation indicates that the distribution of individuals adopting a strategy in degree ranking fundamentally changes the frequency of the strategy. In the divide-and-conquer gaming networks, our result suggests that the connectivity in social networks and strategy are two key factors that govern the evolution of the population.

## Introduction

Two-player iterated game is a traditional framework for investigating sentient and evolutionary behaviors, especially for exploring the origin of the emergence of cooperation. In realistic competing scenarios, for instance, in social networks^1–4^, the number of players is usually much more than two, and their composition is also not so well-mixed^5–7^. Based on such a common sense, researchers begin to look for a more proper theoretical framework. In 1992, Nowak and May proposed a ‘one-individual-one-strategy’ framework^8^. In the model, an individual can choose a unified move, cooperation or defection, to play with all his/her neighbors. The system evolves with a learning mechanism that each adopts the winner’s move in the previous round of the game as his/her move in this round. The winner is the one, who gains the highest payoff from the two-player games among the individual and his/her neighbors in the previous round.

In the past decade, the learning mechanism has many variants^8–26^, which, to some extent, explains the widespread cooperative behaviors in social networks. The extent is limited by the assumption ‘one-individual-one-strategy’. In the hypothesis, an individual has to choose a unified move to play with neighbors, while humans do not normally change their moves to one opponent because of their gaming experience with another opponent. For instance, an individual *A* can hardly defect a long-term partner *B* because of *C*’s defection last round. To remedy the drawback, Wardil *et al*. proposed a game model in which an individual simultaneously adopts different strategies against different opponents^27^. In the model, the strategy updating rule is based on the individually accumulated payoffs of the two players on a link. The model provides a higher degree of freedom for the individuals, while does not completely solve the problem. Recalling the example mentioned above, if *A*’s accumulated payoff is greater than *C*, *A* will cooperate with *C* in this round, which seems to be a bit irrational. To better tackle the problem, Zhang *et al*. proposed a divide-and-conquer game model^28^, in which individuals play completely independent games with their neighbors. In the model, two moves are available for selection, to cooperate or to defect. To each neighbor, an individual adopts a specific move. For all the neighbors, therefore, the individual has a pure move set, which is the same as Wardil’s model. The difference is that each pair of the connected individuals plays a two-player infinitely iterated game. The model sets all the individuals to adopt the same strategy to guarantee that they are equally intelligent. Since the focal topic of the evolutionary game theory is to determine whether a strategy will prevail in a population^6,29–33^. A question naturally arises: which strategy will prevail in the social divide-and-conquer gaming networks?

In a structured population, to answer this question is indeed challenging, since strategies alone can hardly determine their prevalence, no matter whether an individual’s fitness is measured by the accumulated payoff or average payoff. A dominant evolutionary strategy in the unstructured population may be dominated in social networks if the individuals adopting this strategy do not possess enough social capital^28,34–37^. In social networks, an individual’s social capital can be represented by a topological property, for instance, his/her connectivity. To fill this gap, we investigate a divide-and-conquer tournament among 14 well-known strategies on social gaming networks. In the tournament, we measure a strategy’s fitness by its player’s accumulated payoff and average payoff, respectively. On the base of an individual’s fitness, the evolution of the population follows a local learning mechanism^8^. We will show that the effect of social capital is determined by ways of measuring fitness. When an individual’s fitness is measured by the accumulated payoff, highly-connected individuals’ strategy is likely to propagate in the system, while less-connected individuals may exhibit polarized fitness when it is measured by the average payoff.

## Results

### Divide-and-conquer game in social networks

If a population of players is well-mixed, that is, a homogeneous population with unbiased random matching, replicator dynamics^38,39^ can provide a good qualitative description of natural selection. Once the organization of the population presents a stable structure, the mean-field assumption will not stand anymore. In this case, researchers turn to agent-based game models^8^. In the past decade, the agent-based games in graphs have been extensively investigated^8–16,33,35–37,40,41^.

**Figure 1:**
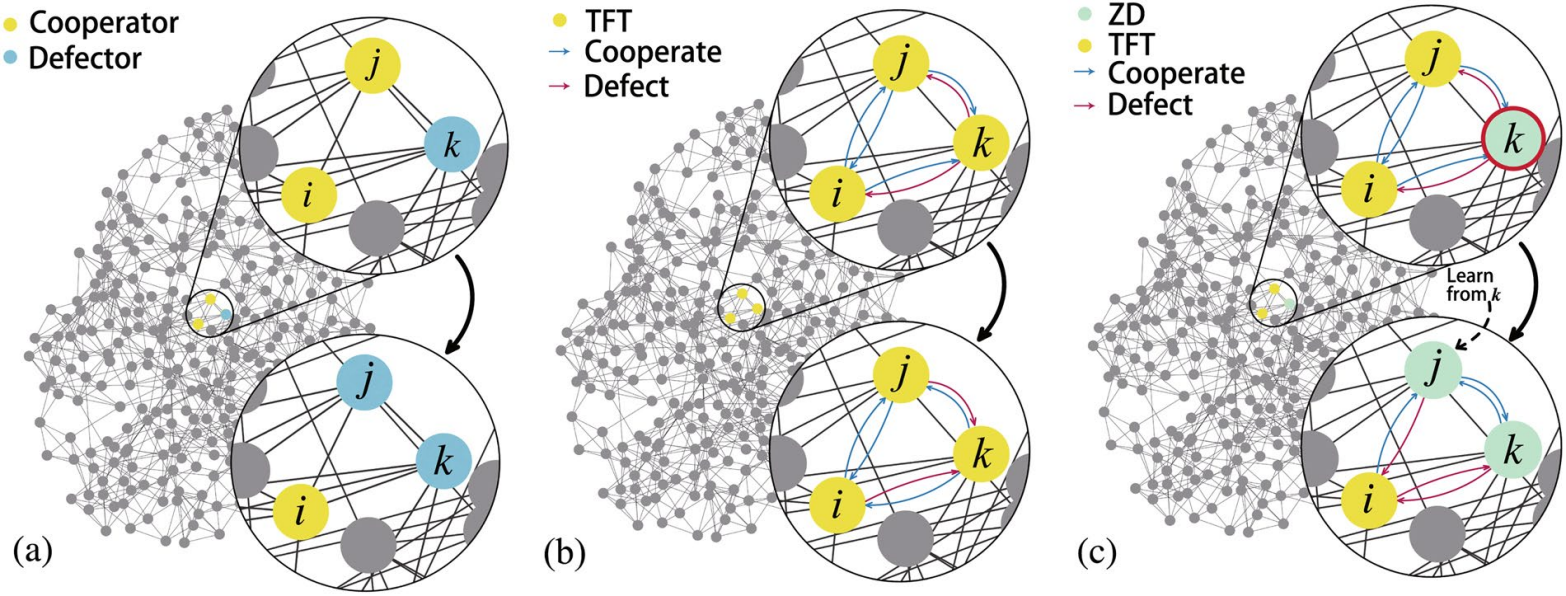
Illustration of three types of evolutionary games in social networks. (**a**) Shows one step of evolution in ‘one-individual-one-strategy’ evolutionary game on a network, where individuals can only choose one unified move to play with all his/her neighbors at each time step. (**b**) Shows one step of evolution in a divide-and-conquer game, where all the individuals follow an identical strategy. Each individual updates his/her move specified to a neighbor based on his/her gaming experience with the neighbor in the past rounds. (**c**) Shows one step of evolution in a match between two strategies in a divide-and-conquer game, where the update of strategy is based on a local learning mechanism. *j* will copy *k*’s strategy (in the red circle), since *k* is the one gaining the highest fitness in *j*’s neighborhood in the past a number of rounds. In this figure, black arrows denote one step of evolution, where their tails connect with the statuses before evolving.

In the agent-based games, researchers usually assume that all players use the same strategy, which is typically called ‘strategy updating rule’ in the previous studies. The strategy updating rules are proposed to mimic Darwinian selection or human learning with a bounded rationality. When games are played on a graph or network, a player may not only update its move but also break its connections to neighbors. Here, the player can only choose one move, cooperation (C) or defection (D), to play with all its neighbors as shown in Fig. 1(a). However, s/he can choose a specific move to play with each neighbor in the divide-and-conquer model as shown in Fig. 1(b). Intuitively, this framework is more suitable for describing realistic competition in social networks.

In this paper, we investigate which strategy can perform better in the divide-and-conquer games. No matter whether in a structured population or a well-mixed population, this is what the researchers who study evolutionary game theory highly concern. We initially assign a strategy to half of a networked population and another strategy to the rest. The individuals start gaming with a cooperative move unless their strategies specify the first move. In each time step, individual plays a two-player two-strategy game with every his/her neighbor. After a round of the game, s/he will receive a payoff from each game. Regarding his/her strategies’ fitness, we discuss two measurements, *ϕ* and *ψ*. Here we define *ϕ* as

1 $${\varphi }_{i}=\sum _{j\in {N}_{i}}\sum _{r\mathrm{=1}}^{ {\mathcal R} }{{\mathscr{P}}}_{ijr},$$

where *N*_*i*_ is the set of *i*’s neighbors, ${\mathcal R}$ is the number of rounds before an individual update his/her strategy, and ${{\mathscr{P}}}_{ijr}$ is *i*’s payoff against *j* in round *r*. We define *ψ* as

2 $${\psi }_{i}=\frac{{\varphi }_{i}}{{k}_{i}},$$

where *k*_*i*_ is the number of *i*’s neighbors.

For individuals, their update of move follows their respective strategies. Their update of strategy is based on a local learning mechanism. Note that in the previous studies on games in structured populations, researchers focus on the update of moves, while the update of strategy has rarely been involved^8,9^. In unstructured populations, researchers typically investigate the update of strategies under the framework of replicator dynamics. In a well-mixed population, there is no stable game relationship between individuals. In social networks, the stable relationship makes the mean-field assumption not stand anymore. Thus, replicator dynamics is not applicable to structured populations. In divide-and-conquer games, every individual has his/her strategy. To tell which strategy can perform better, a game is required to be played for a number of rounds. Here we follow Axelrod’s configuration, setting ℛ to 200, that is, an individual will update his/her strategy after 200 rounds of games. After 200 rounds of games, individual will compare his/her fitness with the highest fitness of his/her neighbors. If his/her fitness is the highest among the neighborhood, the individual will keep his/her strategy. Otherwise, s/he will copy the winner’s strategy.

Notably, after updating strategies, the first move of an individual will depend on previous gaming history. For example, there are two strategies in a structured population, AD and Tit-For-Tat (TFT). An AD player always defect, while a TFT player copies his/her opponent’s previous move. We focus on two AD players in this population, ${\mathscr{A}}$ and ${\mathscr{B}}$. After 200 rounds, ${\mathscr{B}}$ updates to a TFT player because a TFT player in the neighborhood exhibits the highest fitness. According to the definition, the first move of a TFT player is to cooperate. However, since ${\mathscr{A}}$ defects in the previous round, the new TFT player ${\mathscr{B}}$ will defect at the first round after updating its strategy.

In a word, we assign two strategies to a structured population. Individuals update their strategies according to a local learning mechanism after a number of rounds. In this way, we can test the fitness of the two strategies in a structured population, while to find the fittest strategy in the 14 strategies, a tournament is required.

### Divide-And-Conquer Tournament

In a structured population, such as the spatial networks^8,33,35,42^, lattice^33^, random graphs (network), Watts and Strogatz small-world networks (WSSN)^43^, and Barabási and Albert scale-free networks (BASN)^44^, an individual normally has to play with more than one opponent if his/her degree is greater than one. In other words, the individual needs to play more than one game in one round. Considering the different experience gained from the game with various neighbors in the previous rounds, his/her move to the different neighbor should be specific. If the individual only adopts one strategy, the iterated game in networks is defined as a divide-and-conquer game^28^. Since links among individuals are relatively stable in the whole evolution process, individuals are able to play iterated two-player two-strategy games with his/her neighbors.

**Figure 2:**
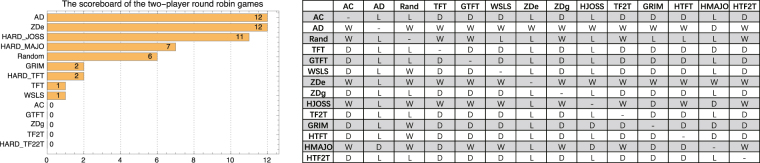
The scoreboard of the two-player round robin games. The table shows the results of the strategies in the column versus the strategies in the row. In the table, ‘W’, ‘L’, ‘D’ denotes ‘win’, ‘lose’, ‘draw’, respectively.

To clarify the influence of network structures on the evolution of strategies, we design a divide-and-conquer tournament on BASN and WSSN. First, we adopt a two-player round-robin game among the 14 strategies to evaluate the fitness of these strategies against each other. The scoreboard is shown in Fig. 2. Each game is a 10, 000-round 2 × 2 game repeated 100 times. The strategy which gains a higher aggregated payoff wins. If both of the two strategies in a game gain the same payoff, the strategy which gains a higher payoff playing with itself wins. If the two strategies gain the same payoff playing with themselves, the game is a draw. For each two strategies, unless their payoffs gained from playing with each other and with themselves are all the same, they will be chosen for the divide-and-conquer tournament. For convenience, we call the strategy losing the game the weak strategy and the other the strong strategy. In divide-and-conquer tournament, the initial numbers of the individuals adopting the respective strategy are the same. We define *α* as

3 $$\varepsilon =\lfloor \alpha \mu \rfloor ,$$

where *ε* is the number of the chosen weak strategy players, and *μ* is the initial number of the weak players in the network. Here we test a series of values of *α*, ranging from 0 to 1, with the step size of 0.01. Assign *ε* weak strategy players to the top nodes in the degree sequence in descending order. After every 200 rounds, individuals will update their strategies following the local learning mechanism mentioned in the previous section.

For each match with a certain *α*, we run 20, 000 rounds of the IPD game, including 100 updates of strategies at maximum. We test 101 values of *α*, each of which is implemented for ten times. Thus, we run 20, 200, 000 rounds of the IPD game for a match between two strategies. Cutting the draw matches, we hold 54 matches for a certain network and a certain measurement of fitness. In total, we run 8, 726, 400, 000 rounds of the IPD game. In our experiment, we shape the structure of individuals into two BASN and two WSSN. The tested BASN are composed of 3, 066 and 6, 135 links, which means that each round of the IPD game includes 3, 066 and 6, 135 PD games, respectively. For the WSSN in the tournament, they are both formed by 3, 072 links, while the values of *p* are 0.1 and 0.2, respectively.

For the convenience of clarifying the experimental results, we denote the expected payoff of strategy *S*_*a*_ playing against strategy *S*_*b*_ in the stationary state by *E*(*S*_*a*_, *S*_*b*_). We define *δ*(*S*_*a*_, *S*_*b*_)  = *E*(*S*_*a*_, *S*_*a*_) − *E*(*S*_*b*_, *S*_*a*_). We denote the weak strategy by *S*_*w*_ and the strong strategy by *S*_*s*_, respectively. In this paper, for a certain match between two strategies, we uniformly assign the weak strategy before the strong one. For example, for a match of AC versus AD, AC is the weak strategy in the match.

### Fitness is measured by *ψ*

**Figure 3:**
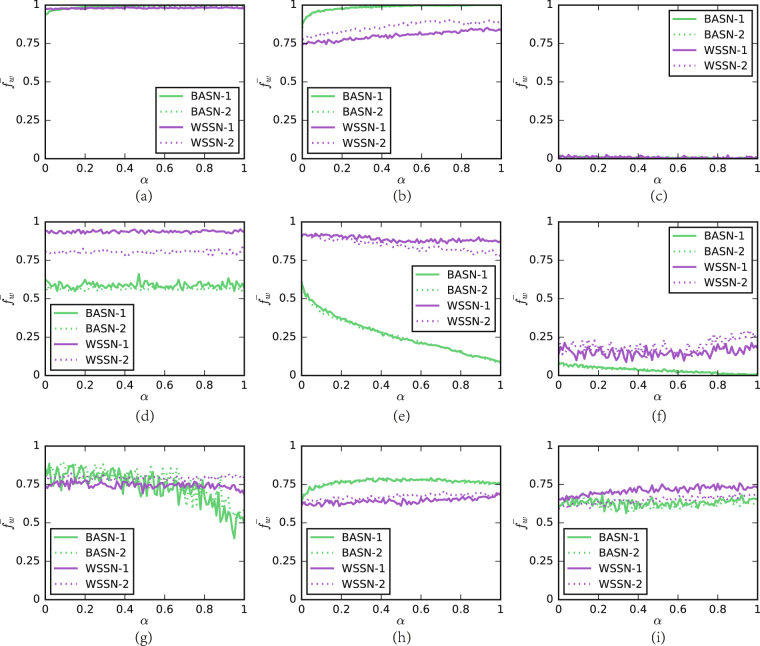
The results of matches adopting measurement *ψ*. ${\bar{f}}_{w}$ is the frequency of the weak strategy. *α* is the percentage of weak strategists to be assigned to the top of degree sequences. We generate two WSSN with *N* = 1024, *k* = 6, and *p* = 0.1 for WSSN-1, *N* = 1024, *k* = 6, and *p* = 0.2 for WSSN-2, and two BASN with *m* = *m*_0_ = 3 and *N* = 1024 for BASN-1, *m* = *m*_0_ = 3 and *N* = 2048 for BASN-2. (**a**) Shows the result of the match between TFT and ZDe. (**b**) Shows the result of the match between HARD_TFT and HARD_JOSS. (**c**) Shows the result of the match between Random and AD. (**d**) Shows the result of the match between Random and ZDe. (**e**) Shows the result of the match between TF2T and HARD_MAJO. (**f**) Shows the result of the match between TF2T and Random. (**g**) Shows the result of the match between WSLS and AD. (**h**) Shows the result of the match between ZDe and AD. (**i**) Shows the result of the match between Random and HARD_MAJO.

Figure 3 shows the result of the case that individual’s fitness is measured by the average payoff *ψ*. We test a series of values of *α* to investigate the influence of individuals’ connectivity on the frequency of strategy in the stable state. Since for some matches, the results are similar to a large extent. For these matches, we show a representative example here. When fitness is measured by the average payoff, social structure has subtle influence on the evolution of strategies. Generally, one can observe that the measurement of fitness considerably limits the impact of *α* on ${\bar{f}}_{w}$. No matter whether ${\bar{f}}_{w}$ grows or decays with *α*, the slope is relatively small. Nevertheless, the impact is detectable. In the sense of mean-field approximation, average payoff of each individual should be identical in a long run if they adopt the same strategy. In this case, permuting the location of individuals with different degrees in a network would not fundamentally change their fitness. However, our previous results suggest that individuals with less connectivity are more likely to be unfavorable individuals, who gain the lowest average payoff in a round of game^28^. Instead, the fitness of highly-connected individuals are rather stable. Thus, the growth of *α* promotes the robustness of the weak strategists, which brings them a modest advantage. The advantage is typically emerges with the growth of ${\bar{f}}_{w}$. Meanwhile, one should notice the polarization of the fitness of the less-connected individuals^28^. They are also more likely to receive the highest average payoff, causing that strong strategy can hardly be eliminated in the network. In order to test the scalability of the conclusion, we run our experiments on a series of networks with different size. Interestingly, the results are almost the same, indicating that the impact of the size on the evolution is negligible. In the WSSN, on the other hand, a larger *p* means a more degree-heterogeneous network. Thus, for most matches, the curves of the WSSN-2 (*p *= 0.2) generally drifts to the BASN to a small extent.

Figure 3(a) shows the result of the match of TFT versus Zero-Determinant Extortionate strategy (ZDe)^31,40,41^, which is similar to the results of the matches of TFT versus HARD_JOSS^45^, TFT versus AD, Tit-For-two-Tat (TF2T)^31^ versus ZDe, TF2T versus AD, HARD_TFT^31^ versus ZDe, HARD_TFT versus AD, HARD_TF2T^31^ versus ZDe, HARD_TF2T versus Random, HARD_TF2T versus AD, HARD_JOSS versus ZDe, HARD_JOSS versus AD, Grim trigger (GRIM)^46^ versus ZDe, and GRIM versus AD. In Fig. 3(a), one can observe that the frequency of the weak strategy ${\bar{f}}_{w}$ is close to 94% in the BASN and 97% in the WSSN for *α* = 0, respectively. In the BASN, ${\bar{f}}_{w}$ grows slowly with *α*, while it is almost irrelevant to *α* in the WSSN. For *α* = 100%, ${\bar{f}}_{w}\approx \mathrm{98 \% }$ in the WSSN, which indicates the weak strategy can hardly wipe out the strong strategy. This is because the weak strategy can not further invade the cluster of the strong strategy, since less-connected strong strategy players are as well possible to receive the highest average payoff when they are surrounded by the weak strategists. In all these matches, *δ*(*S*_*w*_, *S*_*s*_) are greater than *δ*(*S*_*s*_, *S*_*w*_), thus the weak strategy can always occupy a larger fraction of the population.

Figure 3(b) shows the result of the match of HARD_TFT versus HARD_JOSS, which is similar to the result of the match of GRIM versus HARD_JOSS. In the BASN, the rate of growth for ${\bar{f}}_{w}$ is slightly higher than that in Fig. 3(a). In the WSSN, it grows slowly with *α*. The growth of ${\bar{f}}_{w}$ confirms the impact from the promotion of robustness on the fitness of the weak strategists mentioned above.

Figure 3(c) shows the result of the match of Random versus AD, which is similar to the results of the matches of Random versus HARD_TFT, Random versus HARD_JOSS, and Hard Majority (HARD_MAJO)^31^ versus GRIM. In Fig. 3(c), one can observe that ${\bar{f}}_{w}$ is approximately equal to 0% in both the BASN and WSSN for *α* = 0, respectively. With the growth of *α*, ${\bar{f}}_{w}$ remains close to 0%, owing to that *δ*(*S*_*w*_, *S*_*s*_) are much less than *δ*(*S*_*s*_, *S*_*w*_). For example, *δ*(*Random*, *AD*) =  −0.75, while *δ*(*AD*, *Random*) = 0.5. Since the weak strategy is not strong enough, even it is adopted by nodes with a large degree, it can hardly survive in the fierce competition.

Figure 3(d) shows the result of the match of Random versus ZDe, which is similar to the results of the matches of HARD_TF2T versus HARD_JOSS, AC versus ZDe, AC versus HARD_JOSS, and TF2T versus HARD_JOSS. In Fig. 3(d), ${\bar{f}}_{w}$ is, again, irrelevant to *α*. The reason is, for the two strategies in these matches, *δ*(*S*_*w*_, *S*_*s*_) and *δ*(*S*_*s*_, *S*_*w*_) are close to each other. Take the match between Random and ZDe for example, in which *δ*(*Random*, *ZDe*) =  −0.21 and *δ*(*ZDe*, *Random*) =  −0.52, the distance between *δ*(*Random*, *ZDe*) and *δ*(*ZDe*, *Random*) is merely 0.31. In this case, one can see that assigning weak strategy players to the top nodes in the degree sequence can not significantly promote ${\bar{f}}_{w}$.

Figure 3(e) shows the result of the match of TF2T versus HARD_MAJO, which is similar to the results of the matches of Zero-Determinant strategy generous version (ZDg)^31^ versus HARD_MAJO, HARD_TF2T and HARD_MAJO, Generous Tit-For-Tat (GTFT) versus HARD_MAJO, and AC versus HARD_MAJO. In Fig. 3(e), one can observe that ${\bar{f}}_{w}$ decays with *α* both in the BASN and WSSN, while the rate in the BASN is much higher. In these matches, the strong strategy is HARD_MAJO which begins with defection and the initial moves of the weak strategists are uniformly cooperation. At the beginning, a HARD_MAJO player gets a higher payoff when playing with a weak strategist but suffers from the punishment of mutual defection when playing with a HARD_MAJO player. After a weak strategist updates his/her strategy to HARD_MAJO, s/he will cooperate with his/her neighbors, since the majority of their previous moves are cooperation. In this case, with the growth of *α*, the probability of the weak strategists connecting with HARD_MAJO players increases at the beginning. The result is that HARM_MAJO is able to occupy more individuals after 200 rounds, leading to the decay of ${\bar{f}}_{w}$.

Figure 3(f) shows the result of the match of TF2T versus Random, which is similar to the result of the match of HARD_MAJO versus ZDe. In these matches, *δ*(*S*_*w*_, *S*_*s*_) are less than *δ*(*S*_*s*_, *S*_*w*_). Thus, the frequency of the weak strategy is restricted. In the WSSN, ${\bar{f}}_{w}$ grows slightly with *α*, while in the BASN, it decays with *α*. The reason is that with the growth of *α*, the probability of the weak strategists connecting with the strong strategists rises, which makes the weak strategists harder to survive. Since the WSSN is degree-homogeneous, the influence is negligible.

Figure 3(g) shows the result of the match of WSLS versus AD. The weak strategy occupies a larger fraction of the population than which of Fig. 3(f), since *δ*(*S*_*w*_, *S*_*s*_) is greater than *δ*(*S*_*s*_, *S*_*w*_). One can observe that in the BASN, ${\bar{f}}_{w}$ fluctuates dramatically with *α*. The behavior results from the system are sensitive to the initial assignment of the weak strategy players, especially to their positions in the network.

Figure 3(h) shows the result of the match of ZDe versus AD, which is similar to the results of the matches of WSLS versus HARD_MAJO. In Fig. 3(h), ${\bar{f}}_{w}$ grows with *α* both in the BASN and WSSN, and the rate of growth is close to which of Fig. 3(b). Figure 3(g) shows the result of the match of Random versus HARD_MAJO, in which ${\bar{f}}_{w}$ changes slightly with *α* for the two networks. Since HARD_MAJO imitates the major move of the opponent, a HARD_MAJO player will act as a Random player when the opponent is a Random player. Thus, the influence of strategies can be neglect after several updates. As a result, one can observe the modest advantage on the robustness for the weak strategists is promoted by the growing *α*.

Besides, our simulation result shows that the weak strategy, in some matches, wipes out the strong strategy for all the values of *α* ranging from 0 to 1. In the case that the fitness is measured by *ψ*, the behavior is observed in the matches of GTFT versus AD, GTFT versus HARD_JOSS, GTFT versus Random, GTFT versus ZDe, TFT versus Random, WSLS versus HARD_JOSS, WSLS versus ZDe, ZDg versus AD, ZDg versus HARD_JOSS, ZDg versus Random, and ZDg versus ZDe. Meanwhile, there are several matches where the weak strategy can not survive for *α* ranging from 0 to 1. We observe this behavior in the matches of AC versus AD, AC versus Random, HARD_MAJO versus HARD_JOSS, HARD_MAJO versus HARD_TFT, HARD_MAJO versus TFT, Random versus WSLS, and Random versus GRIM. In these matches, the influence of strategies overwhelms that of network structures.

### Fitness is measured by *ϕ*

**Figure 4:**
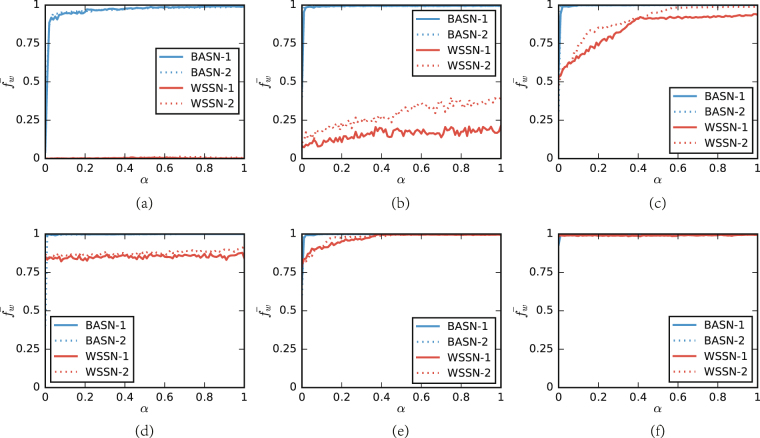
The results of matches adopting measurement *ϕ*. ${\bar{f}}_{w}$ is the frequency of the weak strategy. *α* is the percentage of weak strategist to be assigned to the top of degree sequences. We generate two WSSN with *N* = 1024, *k* = 6, and *p* = 0.1 for WSSN-1, *N* = 1024, *k* = 6, and *p* = 0.2 for WSSN-2, and two BASN with *m* = *m*_0_ = 3 and *N* = 1024 for BASN-1, *m* = *m*_0_ = 3 and *N* = 2048 for BASN-2. (**a**) Shows the result of the match between HARD_MAJO and TFT. (**b**) Shows the result of the match between HARD_MAJO and ZDe. (**c**) Shows the result of the match between ZDe and AD. (**d**) Shows the result of the match between WSLS and AD. (**e**) shows the result of the match between GTFT and HARD_JOSS. (**f**) Shows the result of the match between HARD_T2FT and ZDe.

Figure 4 shows the result of the case that individual’s fitness is measured by the accumulated payoff *ϕ*. We test a series of values of *α* to investigate the influence of individuals’ degree on the frequency of strategy in the stable state. Since for some matches, the results are similar to a large extent. For these matches, we show a representative example here. We will show that the impact from topological structures can fundamentally leverage the balance of the matches.

To precisely clarify the impact from individual degree, we briefly introduce how to calculate the fitness of an individual in networks. Individual *i*’s fitness at the *n* ^*th*^ round can be written as^17^:

4 $${G}_{i}(n)={k}_{i}({{\rm{\Delta }}}_{i}(n){X}_{i}(n)+T{W}_{i}(n)+P\mathrm{(1}-{W}_{i}(n))),$$

where *k*_*i*_ denotes the individual *i*’s degree. *X*_*i*_(*n*) denotes *i*’s move at the *n* ^*th*^ round, where *X*_*i*_(*n*) = 1 if *i* cooperates, otherwise *X*_*i*_(*n*) = 0. Δ_*i*_(*n*) = *S* − *P* + (*R* − *T* + *P* − *S*)*W*_*i*_(*n*), where *W*_*i*_(*n*) denotes *i*’s local frequency of cooperators at the *n* ^*th*^ round, and *R*, *T*, *S*, *P* are the four parameters of PD game (see section Methods). From the equation, one can see that the impact of individual’s connectivity on the fitness is much greater than the influence of strategies. Generally, for the BASN, the weak strategy can propagate, even dominate the network, when occupy the hubs of the networks. For the WSSN, an interesting observation is that in a WSSN with a larger *p*, the weak strategy can occupy more individuals. The behavior indicates that the impact of the accumulated payoff on the competition between strategies grows with the degree heterogeneity.

Figure 4(a) shows the result of the match of HARD_MAJO versus TFT, which is similar to the results of the matches of AC versus AD, HARD_MAJO versus GRIM, Random versus GRIM, and Random versus WSLS. In Fig. 4(a), one can observe that ${\bar{f}}_{w}$ is approximately equal to 4% in the BASN and 0% in the WSSN for *α* = 0, respectively. In the BASN, ${\bar{f}}_{w}$ drastically grows to 92% when *α* reaches 3%, after which it grows slowly with *α* and approaches 100%. The observation indicates that the strong degree heterogeneity of the BASN brings a hub or highly-connected individual a remarkable advantage, which properly remedies the drawback of his/her strategy. In the WSSN, ${\bar{f}}_{w}$ is basically irrelevant to *α*. The result indicates that the advantage brought by the connectivity is not strong enough. The weak strategists are too weak to invade the cluster of the strong strategy, which are generally struggling at the edge of elimination.

Figure 4(b) shows the result of the match of HARD_MAJO versus ZDe, which is similar to the results of the matches of AC versus Random, HARD_MAJO versus HARD_JOSS, HARD_MAJO versus HARD_TFT, Random versus AD, and Random versus HARD_TFT. In Fig. 4(b), one can observe that in both BASN and WSSN, ${\bar{f}}_{w}$ are small when *α* = 0, since *δ*(*S*_*w*_, *S*_*s*_) is less than *δ*(*S*_*s*_, *S*_*w*_). After that, ${\bar{f}}_{w}$ in the BASN jumps to around 95% when *α* reaches 1%, while grows slowly in the WSSN. The reason has been provided in Fig. 3(a).

Figure 4(c) shows the result of the match of ZDe versus AD, which is similar to the results of the matches of AC versus HARD_JOSS, GRIM versus HARD_JOSS, HARD_TF2T versus HARD_JOSS, HARD_TF2T versus Random, HARD_TFT versus HARD_JOSS, Random versus HARD_JOSS, Random versus HARD_MAJO, TF2T versus HARD_JOSS, and TF2T versus Random. In Fig. 4(c), the jump of ${\bar{f}}_{w}$ is similar to that in Fig. 4(b). In the WSSN, ${\bar{f}}_{w}$ grows with *α*, and stops growing after *α* reaches 40% in WSSN-1 and 60% in WSSN-2, respectively. For the WSSN, the degree homogeneity limits the impact from the topological structures. Thus, only a small part of weak strategists benefits from the growth of connectivity. When *α* reaches a certain value, the number of the benefited individuals gets its maximum.

Figure 4(d) shows the result of the match of WSLS versus AD, which is similar to the results of the matches of AC versus ZDe and WSLS versus HARD_MAJO. For the BASN, Fig. 4(d) shows a similar result to Fig. 4(c). For the WSSN, the behavior and its origin are similar to Fig. 3(d). Although the distance between the competing strategies is enhanced by the measurement of fitness, the distance is likewise too weak to govern the evolution of the system.

Figure 4(e) shows the result of the match of GTFT versus HARD_JOSS, which is similar to the results of the matches of AC versus HARD_MAJO, GTFT versus HARD_MAJO, GTFT versus Random, HARD_TF2T versus HARD_MAJO, Random versus ZDe, TF2T versus HARD_MAJO, TFT versus HARD_JOSS, TFT versus Random, ZDg versus HARD_JOSS, and ZDg versus Random. Figure 4(e) shows a similar result as Fig. 4(c). The only difference is that the value of ${\bar{f}}_{w}$ in Fig. 4(e) is greater than that in Fig. 4(c), since the distance between *δ*(*S*_*w*_, *S*_*s*_) and *δ*(*S*_*s*_, *S*_*w*_) is larger.

Figure 4(f) shows the result of the match of HARD_T2FT versus ZDe, which is similar to the results of the matches of GRIM versus AD, GRIM versus ZDe, GTFT versus AD, GTFT versus ZDe, HARD_JOSS versus AD, HARD_JOSS versus ZDe, HARD_TF2T versus AD, HARD_TFT versus AD, HARD_TFT versus ZDe, TF2T versus AD, TF2T versus ZDe, TFT versus AD, TFT versus ZDe, WSLS versus HARD_JOSS, and ZDg versus HARD_MAJO. In the BASN, ${\bar{f}}_{w}$ immediately grows to 100% when *α* reaches 1%. In the WSSN, ${\bar{f}}_{w}$ grows slowly with *α*. In these matches, the strong strategists tend to defect themselves, that is, *W*_*i*_ is very small. So is their fitness. Meanwhile, the distance between *E*(*S*_*w*_, *S*_*s*_) and *E*(*S*_*s*_, *S*_*w*_) is negligible, which indicates that advantage of the strong strategy is not so significant. Thus, even when the strategists directly connect with the weak strategists, they can not receive a much higher payoff to remedy the disadvantage on their payoff from mutual defection. Therefore, the strong strategies are almost wiped out in these matches.

Besides, our simulation result shows that the weak strategy, in some matches, wipes out the strong strategy for all the values of *α* ranging from 0 to 1. The matches are ZDg versus ZDe, ZDg versus AD, and WSLS versus ZDe.

### The influence of strategy

In our tournament, for the case of *α* = 0, i.e., two strategies are randomly assigned to the individuals in the network, the influence of structures on the system is very limited. In the extreme case, the fitness of the strategies is totally determined by themselves.

Generally, if *δ*(*S*_*w*_, *S*_*s*_) is much less than *δ*(*S*_*s*_, *S*_*w*_), weak strategies can hardly survive in the population. In Fig. 4(a), HARD_MAJO as a weak strategy plays with TFT, *δ*(*S*_*w*_, *S*_*s*_) =  −1.5 and *δ*(*S*_*s*_, *S*_*w*_) = 0.5. Clearly, HARD_MAJO can not find a foothold in the stable state in both fitness measurements. A similar result can be found in Fig. 3(c).

If *δ*(*S*_*w*_, *S*_*s*_) is less than *δ*(*S*_*s*_, *S*_*w*_), but the distance is limited, the frequency of weak strategies is restricted. Figure 3(f) shows the result of matches between TF2T and Random where *δ*(*S*_*w*_, *S*_*s*_) =  −0.125 and *δ*(*S*_*s*_, *S*_*w*_) = 0.375. In the case, the weak strategy can survive. Precisely, ${\bar{f}}_{w}$ is approximately equal to 7% in the BASN and 14% in the WSSN, respectively. Figure 4(b) shows the result of matches between HARD_MAJO and ZDe, where *δ*(*S*_*w*_, *S*_*s*_) =  −1 and *δ*(*S*_*s*_, *S*_*w*_) =  −0.34 and ${\bar{f}}_{w}$ is approximately equal to 40% in the BASN and 9% in the WSSN, respectively. Note that ${\bar{f}}_{w}$ doesn’t monotonously grows with the distance between *δ*(*S*_*w*_, *S*_*s*_) and *δ*(*S*_*s*_, *S*_*w*_), in that individuals adopt the local learning mechanism, which is the best-takes-over^8^. In other words, for an individual, gaming with a neighbor with a bit higher fitness than the other neighbors is equal to gaming with the neighbor with a much higher fitness.

If *δ*(*S*_*w*_, *S*_*s*_) is greater than *δ*(*S*_*s*_, *S*_*w*_), meanwhile $\delta ({S}_{s},{S}_{w})\geqslant 0$ or $0\geqslant \delta ({S}_{w},{S}_{s}) > \delta ({S}_{s},{S}_{w})$, weak strategies can occupy a larger fraction of populations. Figure 3(b) show the result of matches between HARD_TFT and HARD_JOSS. For these two strategies, *δ*(*S*_*w*_, *S*_*s*_) = 2 and *δ*(*S*_*s*_, *S*_*w*_) = 1.35. Clearly, HARD_TFT occupies more than 50% of the population. The reason is similar to that of Fig. 3(a)(d)(e)(i) and Fig. 4(e)(f).

If *δ*(*S*_*w*_, *S*_*s*_) is greater than *δ*(*S*_*s*_, *S*_*w*_) and *δ*(*S*_*w*_, *S*_*s*_) 0 *δ*(*S*_*s*_, *S*_*w*_), weak strategies can eliminate strong strategies. For example, in the match between ZDg and ZDe with *δ*(*S*_*w*_, *S*_*s*_) = 0.5 and *δ*(*S*_*s*_, *S*_*w*_) =  −0.5. In this case, weak strategies can eliminate strong strategies for all the values of *α* ranging from 0 to 1.

### Influence of topological structures

Overall, we discuss two measurements of fitness, where social structures play different roles. When we adopt the measurement *ψ*, the influence of social structures is less than that of strategies. In both types of networks, a large *α* can not guarantee the prevalence of the weak strategy. Since the WSSN are degree homogeneous, the influence from individual connectivity is relatively modest. In the BASN, although they are degree-heterogeneous networks, owing to the definition of fitness, the influence of connectivity becomes rather subtle, which has been analytically investigated in our previous work^28^. Thus, one can observe that ${\bar{f}}_{w}$ is not so sensitive to *α* in Fig. 3 as what it is in Fig. 4.

As for measurement *ϕ*, the impact of an individual’s degree on his/her fitness is amplified. Thus, topological structures play a significant role in both networks, which is confirmed by the observations in Fig. 4. In the WSSN, the more weak strategists are assigned to the top individuals in the degree sequence, the higher the frequency of the weak strategy will be at the end of the match. In some matches on the BASN, weak strategies unexpectedly dominate the population when *α* is merely 1%. This behavior indicates that the influence of social structures is much stronger than that of strategies.

## Discussions

Divide-and-conquer game provides a theoretical framework for more precisely abstracting realistic interactions among sentient individuals. Individuals will update their moves according to their strategies. Each update of an individual’s move is based on the gaming trajectory with a neighbor in the previous rounds. For memory-one strategy, an individual is just required to consider the previous one round. Since the individuals are initiated with different moves, the response to different neighbor should be specific even when the individuals adopt a unified strategy. For a population with two strategies, the response varies much more widely.

In order to investigate how the divide-and-conquer gaming system evolves under the framework with two strategies, we design a set of tournaments. First, we simulate a two-player two-strategy round-robin tournament. In each match, the winning strategy is referred to as the strong strategy, while the loser is called the weak strategy. Next, we run a divide-and-conquer tournament on two classical network models, the BASN and WSSN, respectively. In our experiment, we test the influence of individual’s connectivity on the fitness of strategies.

When the fitness is measured by the average payoff, the influence of social structures is subtle. Assigning more weak strategists to the well-connected individuals does not guarantee the growth of its frequency. This behavior originates from that the less-connected individuals have polarized fitness while the fitness of highly-connected players is normally close to the average fitness^28^. As a result, weak strategies cannot dominate the network in most cases. In some matches, the weak strategy is even too weak to survive.

When the fitness is measured by the accumulated payoff, the impact of connectivity is highly strengthened. In the degree-heterogeneous BASN, weak strategies are able to invade strong strategies, even dominate the population, once a small part of them are assigned to the highly-connected individuals, even for the case that the strong strategies play well with themselves. In the degree-homogeneous WSSN, the promotion on the fitness of the weak strategists is less, compared with the BASN, but the behavior is likewise observable.

In both cases, our result reveals that strategies are not the only factor governing the evolution of gaming system. Social structures sometimes play an even more critical role in a match between two strategies on networks. Our observations indicate that the number of connections as a sort of social capital, leverages the balance between two strategies to a large extent. We believe that the divide-and-conquer tournament provides a paradigm for investigating the competition between strategies in complex networks. Our results are helpful to further understanding the impact of topological structures on social competitions.

## Methods

### Two-player two-strategy game

In a two-strategy game, we define *i*’s strategy as

5 $${{\rm{\Omega }}}_{i}=(\begin{array}{c}{X}_{i}\\ 1-{X}_{i}\end{array})\mathrm{.}$$

*X*_*i*_ can only take 1 or 0 in each game. For *X*_*i*_ = 1, *i* is a cooperator denoted by *C*. For *X*_*i*_ = 0, *i* is a defector denoted by *D*. We take the Prisoner’s Dilemma (PD)^47,48^ for example. As a heuristic framework, the Prisoner’s Dilemma describes a commonly identified paradigm in many real-world situations. It has been widely studied as a standard model for the confrontation between altruistic and selfish behaviors. The egocentric behavior here is manifested by a defective strategy, aspiring to obtain the greatest benefit from the interaction with others. This PD game model considers two prisoners who are placed in separate cells. Each prisoner must decide to confess (defect) or keep silence (cooperate). A prisoner may receive one of the following four different payoffs depending on both its own strategy and the other prisoner’s strategy. It gains *T* (temptation to defect) for defecting a cooperator, *R* (reward for mutual cooperation) for cooperating with a cooperator, *P* (punishment for mutual defection) for defecting a defector, and *S* (sucker’s payoff) for cooperating with a defector. Normally, the four payoff values satisfy the following inequalities: $T > R > P\geqslant S$ and 2*R* > *T* + *S*. Here, 2*R* > *T* + *S* makes mutual cooperation the best outcome from the prospective of the interest of these two-person group.

In the PD, the payoff table is a 2 × 2 matrix. Given equation ((5)), *i*’s payoff in a game playing with *j* can be written as

6 $${G}_{i}={{{\rm{\Omega }}}_{i}}^{T}(\begin{array}{cc}R & S\\ T & P\end{array})\sum _{j}{{\rm{\Omega }}}_{j}\mathrm{.}$$

In our experiment, the first part is 2 × 2 round robin tournament. To better understand TFT, GTFT, HARD_JOSS, WSLS, ZDe and ZDg strategies in the tournament, we briefly introduce the memory-one strategies in the 2 × 2 iterated game here. For each game between two players, each player has to experience one of the four possible cases, namely, cooperating with a cooperator (CC), cooperating with a defector (CD), defecting a cooperator (DC), and defecting a defector (DD). We define a state vector **Φ** by (Φ_*CC*_, Φ_*CD*_, Φ_*DC*_, Φ_*DD*_), in which each entry corresponds to the probability of experiencing the respective outcome. Generally, a memory-one strategy can be written as ***p*** = (*p*_*CC*_, *p*_*CD*_, *p*_*DC*_, *p*_*DD*_), corresponding to the probabilities of cooperating under each of the previous outcomes. Since players update their moves with the memory-one strategies in each time step, the update can be considered as a Markov process. One can find a Markov transition matrix *M* to realize the update. For two players, *A* and *B*, we have

7 $${M}_{A}=(\begin{array}{cccc}{p}_{CC}{s}_{CC} & {p}_{CC}\mathrm{(1}-{s}_{CC}) & \mathrm{(1}-{p}_{CC}){s}_{CC} & \mathrm{(1}-{p}_{CC}\mathrm{)(1}-{s}_{CC})\\ {p}_{CD}{s}_{DC} & {p}_{CD}\mathrm{(1}-{s}_{DC}) & \mathrm{(1}-{p}_{CD}){s}_{DC} & \mathrm{(1}-{p}_{CD}\mathrm{)(1}-{s}_{DC})\\ {p}_{DC}{s}_{CD} & {p}_{DC}\mathrm{(1}-{s}_{CD}) & \mathrm{(1}-{p}_{DC}){s}_{CD} & \mathrm{(1}-{p}_{DC}\mathrm{)(1}-{s}_{CD})\\ {p}_{DD}{s}_{DD} & {p}_{DD}\mathrm{(1}-{s}_{DD}) & \mathrm{(1}-{p}_{DD}){s}_{DD} & \mathrm{(1}-{p}_{DD}\mathrm{)(1}-{s}_{DD})\end{array}),$$

where the vectors **p** = (*p*_*CC*_, *p*_*CD*_, *p*_*DC*_, *p*_*DD*_) and **s** = (*s*_*CC*_, *s*_*CD*_, *s*_*DC*_, *s*_*DD*_) denote *A* and *B*’s probabilities of cooperation in the next round after experiencing CC, CD, DC, and DD cases, respectively. If *i* is a completely irrational individual with a constant probability of cooperation *q*, **p** = (*q*, *q*, *q*, *q*). Then the evolution of A’s state vector **Φ**_*A*_(*t*) is given by

8 $${{\boldsymbol{\Phi }}}_{A}(t)={{\boldsymbol{\Phi }}}_{A}(t-\mathrm{1)}{M}_{A}\mathrm{.}$$

For the memory-one strategies, their expectation can be calculated by

9 $${E}_{x}=\frac{D(p,s,{{\rm{S}}}_{x})}{D(p,s\mathrm{,1)}}$$

where

10 $$D(p,s,{\bf{f}})=det[\begin{array}{cccc}-1+{p}_{CC}{s}_{CC} & -1+{p}_{CC} & -1+{s}_{CC} & {f}_{1}\\ {p}_{CD}{s}_{DC} & -1+{p}_{CD} & {s}_{DC} & {f}_{2}\\ {p}_{DC}{s}_{CD} & {p}_{DC} & -1+{s}_{CD} & {f}_{3}\\ {p}_{DD}{s}_{DD} & {p}_{DD} & {s}_{DD} & {f}_{4}\end{array}]$$

*p* and *s* here are the same as in Eq. (6), *S*_*x*_ = (*R*, *S*, *T*, *P*) = (3, 0, 5, 1).

In this paper, we denote the expected payoff by *E*(*S*_*a*_, *S*_*b*_). Through comparing the expected payoffs, one can see which strategy is expected to be the strong strategy in a 2 × 2 game. The expected payoff for each 2 × 2 match is shown in Table 1.

**Table 1:** The expected payoff table of the 14 strategies.

	AC	AD	Rand	TFT	GTFT	WSLS	ZDe	ZDg	HJOSS	TF2T	GRIM	HTFT	HMAJO	HTF2T
AC	3	0	1.5	3	3	3	1.91	3	2.7	3	3	3	3	3
AD	5	1	3	1	2.33	3	1	2.23	1	1	1	1	1	1
Rand	4	0.5	2.25	2.25	2.83	2.25	1.52	2.69	2.16	3.125	0.5	0.94	2.25	2.69
TFT	3	1	2.25	3	3	3	1	3	2.5	3	3	3	2.5	3
GTFT	3	0.67	2	3	3	3	1.54	3	2.67	3	3	3	3	3
WSLS	3	0.5	2.25	3	3	3	1.45	3	2	3	3	3	0.5	3
ZDe	3.73	1	2.56	1	2.62	2.36	1	2.5	1	1	1	1	2	1
ZDg	3	0.69	2.06	3	3	3	1.5	3	2.66	3	3	3	3	3
HJOSS	3.2	1	2.29	2.5	3	2	1	2.89	2.35	3.2	1	1	2.5	3.2
TF2T	3	1	1.875	3	3	3	1	3	2.7	3	3	3	3	3
GRIM	3	1	1	3	3	3	1	3	1	3	3	3	1	3
HTFT	3	1	2.81	3	3	3	1	3	1	3	3	3	3	3
HMAJO	3	1	2.25	2.5	3	3	1.34	3	2.5	3	1	3	1	3
HTF2T	3	1	2.06	3	3	3	1	3	2.7	3	3	3	3	3

### The divide-and-conquer game tournament

In the late 1970s, Robert Axelrod conducted a computer tournament^45,49^, in which 14 strategies are submitted to play a round robin game. Let a player represents a strategy. In the game, each player was set to play with the rest of the population, a player with a random strategy and itself. Here, the random strategy means that the player plays *C* and *D* with an identical probability 0.5. Thus, there were 15 opponents for each player. For each match, two players were set to play two hundred rounds of the iterated PD game. Note that the number of rounds is uncertain to the players before the tournament. The entire round robin tournament was repeated for five times to improve the reliability of the results. The entire record of the previous rounds in a match for each pair of players are open to both. All these rules had been announced well before the tournament.

Both Robert Axelrod’s^45,49^ and Stewart’s^31^ tournaments are round robin games between each two strategies. In this paper, we design two tournaments. In our tournaments, 14 strategies are included, which attract most attention in recent studies on game theory^31^. In each match of the first tournament, we run 10, 000 rounds of the iterated PD game. Each match is repeated for 100 times. For each match, we summarize the payoffs of the two strategies and the strategy which gains a higher payoff in total is the winner. The scoreboard of the 14 strategies is shown in Fig. 2.

The second tournament plays in divide-and-conquer gaming networks. In Fig. 2, one can observe which strategy is the winner in a two-player two-strategy game. The winner and loser are called the strong strategy and weak strategy, denoted by *S*_*s*_ and *S*_*w*_, respectively. Specifically, if a graph has *N* nodes, there are initially $\frac{N}{2}$ weak strategy players and $\frac{N}{2}$ strong strategy players. The top $\alpha \times \frac{N}{2}$ individuals of the degree sequence in descending order will be occupied by *S*_*w*_. In the tournament, we test two types of networks, which are the Watts and Strogatz small-world networks (WS)^43^, and Barabási and Albert scale-free networks (BA)^44^. We generate two WSSN with *N* = 1024, *k* = 6, *p* = 0.1 and *N* = 1024, *k* = 6, *p* = 0.2, and two BASN with *N* = 1024, *m* = *m*_0_ = 3 and *N* = 2048, *m* = *m*_0_ = 3. In each round, individual plays a two-player two-strategy game with all his/her neighbors. After the respective games, s/he will receive a payoff. After 200 rounds of games, the fitness of each individual will be measured by the accumulated payoff and average payoff, respectively. Base on the fitness, individuals will update their strategies, following a local learning mechanism^8^. Governed by the local learning mechanism, individual copies the local winner’s strategy. The local winner is one who gains the highest fitness in the neighborhood, including the individual to update his/her strategy. Each match in the tournament will be terminated immediately after extinction of any strategy; otherwise, it will last for at most 20, 000 rounds. The frequency of the weak strategy will be derived by averaging the last 2, 000 rounds if the evolution is not terminated in the middle.

## References

[1] Albert R, Barabási A-L (2002). Statistical mechanics of complex networks. Reviews of modern physics.

[2] Dorogovtsev S, Mendes JFF (2002). Evolution of networks. Adv. Phys..

[3] Newman MEJ (2003). The structure and function of complex networks. SIAM Rev..

[4] Boccaletti S, Latora V, Moreno Y, Chavez M, Hwang DU (2006). Complex networks: Structure and dynamics. Physics reports.

[5] Antal T, Ohtsuki H, Wakeley J, Taylor PD, Nowak MA (2009). Evolution of cooperation by phenotypic similarity. Proceedings of the National Academy of Sciences.

[6] Adami, C. & Hintze, A. Evolutionary instability of zero-determinant strategies demonstrates that winning is not everything. *Nature Communications* **4** (2013). 10.1038/ncomms3193 PMC3741637 23903782

[7] Adami C, Schossau J, Hintze A (2012). Evolution and stability of altruist strategies in microbial games. Physical Review E.

[8] Nowak MA, May RM (1992). Evolutionary games and spatial chaos. Nature.

[9] Santos FC, Pacheco JM (2005). Scale-Free Networks Provide a Unifying Framework for the Emergence of Cooperation. Phys. Rev. Lett..

[10] Santos FC, Rodrigues JF, Pacheco JM (2005). Epidemic spreading and cooperation dynamics on homogeneous small-world networks. Phys. Rev. E.

[11] Kim BJ (2002). Dynamic instabilities induced by asymmetric influence: prisoners’ dilemma game in small-world networks. Physical Review E.

[12] Szolnoki A, Szabó G (2007). Cooperation enhanced by inhomogeneous activity of teaching for evolutionary Prisoner’s Dilemma games. EPL (Europhysics Letters).

[13] Ohtsuki H, Hauert C, Lieberman E, Nowak MA (2006). A simple rule for the evolution of cooperation on graphs. Nature.

[14] Nowak MA (2006). Five rules for the evolution of cooperation. Science (New York, N.y.).

[15] Lieberman E, Hauert C, Nowak MA (2005). Evolutionary dynamics on graphs. Nature.

[16] Gómez-Gardeñes J, Campillo M, Floria LM, Moreno Y (2007). Dynamical organization of cooperation in complex topologies. Physical Review Letters.

[17] Zhang Y, Aziz-Alaoui MA, Bertelle C, Guan J (2014). Local Nash Equilibrium in Social Networks. Scientific Reports.

[18] Zhang Y, Aziz-Alaoui MA, Bertelle C, Zhou S, Wang W (2013). Fence-sitters protect cooperation in complex networks. Phys. Rev. E.

[19] Zhang Y, Aziz-Alaoui MA, Bertelle C, Zhou S, Wang W (2014). Emergence of cooperation in non-scale-free networks. Journal of Physics A: Mathematical and Theoretical.

[20] Gracia-Lázaro C (2012). Heterogeneous networks do not promote cooperation when humans play a Prisoner’s Dilemma. Proceedings of the National Academy of Sciences.

[21] Szabó G, Fáth G (2007). Evolutionary games on graphs. Physics Reports.

[22] Wang H-XY, Rong Z, Wen-Xu (2014). Cooperation percolation in spatial prisoner’s dilemma game. New Journal of Physics.

[23] Szolnoki A, Perc M (2015). Conformity enhances network reciprocity in evolutionary social dilemmas. Journal of The Royal Society Interface.

[24] Szolnoki A, Perc M (2013). Correlation of positive and negative reciprocity fails to confer an evolutionary advantage: Phase transitions to elementary strategies. Physical ReviewX.

[25] Szolnoki A, Perc M (2010). Impact of critical mass on the evolution of cooperation in spatial public goods games. Physical Review E.

[26] Yang H-X, Rong Z (2015). Mutual punishment promotes cooperation in the spatial public goods game. Chaos, Solitons & Fractals.

[27] Wardil L, da Silva JKL (2009). Adoption of simultaneous different strategies against different opponents enhances cooperation. EPL (Europhysics Letters).

[28] Zhang Y, Chen G, Guan J, Zhang Z, Zhou S (2015). Unfavorable Individuals in Social Gaming Networks. Scientific Reports.

[29] Smith, J. M. *Evolution and the Theory of Games* (Cambridge university press, 1982).

[30] Press WH, Dyson FJ (2012). Iterated Prisoner’s Dilemma contains strategies that dominate any evolutionary opponent. Proceedings of the National Academy of Sciences of the United States of America.

[31] Stewart AJ, Plotkin JB (2012). Extortion and cooperation in the Prisoner’s Dilemma. Proceedings of the National Academy of Sciences of the United States of America.

[32] Hao D, Rong Z, Zhou T (2015). Extortion under uncertainty: Zero-determinant strategies in noisy games. Phys. Rev. E.

[33] Perc M, Gomez-Gardenes J, Szolnoki A, Floria LM, Moreno Y (2012). Evolutionary dynamics of group interactions on structured populations: a review. Journal of The Royal Society Interface.

[34] Portes, A. Social capital: Its origins and applications in modern sociology. *LESSER*, *Eric L*. *Knowledge and Social Capital*. *Boston: Butterworth-Heinemann* 43–67 (2000).

[35] Szolnoki A, Perc M (2016). Competition of tolerant strategies in the spatial public goods game. New Journal of Physics.

[36] Szolnoki A, Perc M (2015). Reentrant phase transitions and defensive alliances in social dilemmas with informed strategies. EPL (Europhysics Letters).

[37] Szolnoki A, Chen X (2015). Benefits of tolerance in public goods games. Phys. Rev. E.

[38] Bomze IM (1983). Lotka-Volterra equation and replicator dynamics: a two-dimensional classification. Biological cybernetics.

[39] Zeeman EC (1980). Population dynamics from game theory. Lecture Notes in Mathematics.

[40] Szolnoki A, Perc M (2014). Evolution of extortion in structured populations. Phys. Rev. E.

[41] Szolnoki, A. & Perc, M. Defection and extortion as unexpected catalysts of unconditional cooperation in structured populations. *Scientific Reports* **4** (2014). 10.1038/srep05496 PMC4074784 24975112

[42] Hauert C, Doebeli M (2004). Spatial structure often inhibits the evolution of cooperation in the snowdrift game. Nature.

[43] Watts DJ, Strogatz SH (1998). Collective dynamics of’small-world’networks. Nature.

[44] Barabási A-L, Albert R (1999). Emergence of scaling in random networks. Science (New York, N.y.).

[45] Axelrod R (1980). Effective choice in the prisoner’s dilemma. Journal of Conflict Resolution.

[46] Friedman JW (1971). A non-cooperative equilibrium for supergames. The Review of Economic Studies.

[47] Smith JM (1976). Evolution and the theory of games: in situations characterized by conflict of interest, the best strategy to adopt depends on what others are doing. American Scientist.

[48] Gintis, H. *Game theory evolving: A problem-centered introduction to modeling strategic behavior* (Princeton university press, 2000).

[49] Axelrod R (1980). More effective choice in the prisoner’s dilemma. Journal of Conflict Resolution.

